# Supplementary material for: Identification of photoperiod-induced specific miRNAs in the adrenal glands of Sunite sheep (Ovis aries)
Source: Front Vet Sci. 2022 Jul 22;9:888207. doi: 10.3389/fvets.2022.888207 (PMC9354845; doi:10.3389/fvets.2022.888207)
Supplement: Supplementary file 1 [file Data_Sheet_1.ZIP › Supplementary file/miRDeep2 file.zip!/miRDeep2 file/Know_Structure/SP42A1/oar-mir-21.pdf]

miRBase precursor : oar-mir-21
Total read count : 141838
oar-miR-21 read count : 141827
remaining reads : 11

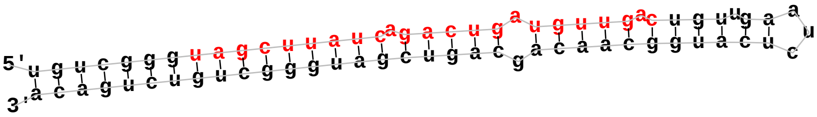

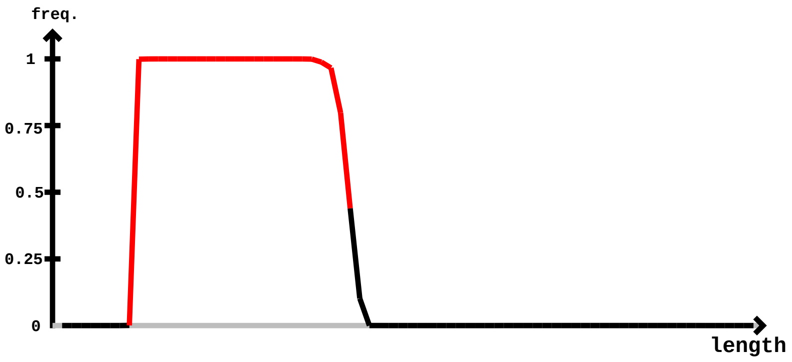


```
            oar-miR-21
5'- ugucggguagcuuaucagacugauguugacuguugaaucucauggcaacagcagucgaugggcugucugaca   -3'    exp
    ((((((((((((((((.(((((.(((((.((((.((...))))))))))).))))))))))))))))))))   reads      mm         sample
    ......guagcuuaucagacugaugu.............................................   1          0          seq
    ......guagcuuaucagacugauguu............................................   6          0          seq
    ......guagcuuaucagacugauguug...........................................   46         0          seq
    ......guagcuuaucagacugauguuga..........................................   19         0          seq
    ......guagcuuaucagacugauguugac.........................................   8          0          seq
    .......uagcuuaucagacugau...............................................   18         0          seq
    .......uagcuuaucagacugaug..............................................   98         0          seq
    .......uagcuuaucagacugaugu.............................................   1580       0          seq
    .......uagcuuaucagacugauguu............................................   3084       0          seq
    .......uagcuuaucagacugauguug...........................................   23784      0          seq
    .......uagcuuaucagacugauguuga..........................................   50810      0          seq
    .......uagcuuaucagacugauguugac.........................................   47831      0          seq
    .......uagcuuaucagacugauguugacu........................................   14377      0          seq
    .......uagcuuaucagacugauguugacug.......................................   5          0          seq
    .......uagcuuaucagacugauguugacugu......................................   6          0          seq
    .......uagcuuaucagacugauguugacuguu.....................................   2          0          seq
    ........agcuuaucagacugaugu.............................................   2          0          seq
    ........agcuuaucagacugauguu............................................   3          0          seq
    ........agcuuaucagacugauguug...........................................   22         0          seq
    ........agcuuaucagacugauguuga..........................................   32         0          seq
    ........agcuuaucagacugauguugac.........................................   45         0          seq
    ........agcuuaucagacugauguugacu........................................   20         0          seq
    .........gcuuaucagacugauguu............................................   1          0          seq
    .........gcuuaucagacugauguug...........................................   5          0          seq
    .........gcuuaucagacugauguuga..........................................   8          0          seq
    .........gcuuaucagacugauguugac.........................................   7          0          seq
    .........gcuuaucagacugauguugacu........................................   2          0          seq
    ..........cuuaucagacugauguugac.........................................   2          0          seq
    ...........uuaucagacugauguuU...........................................   1          1          seq
    ...........uuaucagacugauguuga..........................................   2          0          seq
    ............................................caacagcagucgaugggcugu......   9          0          seq
    ............................................caacagcagucgaugggcugucu....   2          0          seq
```
